# Keystone Veterinary Medical Association

**Published:** 1896-01

**Authors:** 


Secretary.

### KEYSTONE VETERINARY MEDICAL ASSOCIATION.

THIS Association held its regular monthly meeting at Broad and Filbert Streets, Philadelphia, on Tuesday evening, December 10, 1895. President Hart called the meeting to order and Secretary Rhoads called the roll, the following members responding to their names in addition to the officers: Messrs. Goentner, Hoskins, Kooker, Lintz, Pearson, and McAnulty. Dr. George B. Rayner, of Manayunk, was present as a visitor.

Dr. Hoskins, on behalf of the State Board of Examiners, said that at the recent meeting of the Board officers had been elected, the plan of examination considered, and that all applicants would be compelled to undergo an examination, except those who had passed such an examination by Boards of other States, and these would be discretionary with the Board. The first examination would be held at Philadelphia on December 16, 1895.

The failure of the Governor to appoint a State Veterinarian, and thus complete the State Veterinary Sanitary Board, was severely commented upon by a number of those present, and all were urged by the chair to use their influence in obtaining this much-to-be-desired end.

The death of Dr. W. U. Custer, of Reading, was feelingly referred to by Drs. Hoskins and Kooker. After a general discussion of various matters the meeting, at 10.30 P.M., adjourned.

